# THE GENERATIONAL COMPARISONS OF SOCIAL NETWORK SITE USE SUBTYPES AND ITS ASSOCIATIONS WITH SELF-ESTEEM

**DOI:** 10.1093/geroni/igad104.2736

**Published:** 2023-12-21

**Authors:** Bomi Choi, Hayoung Park, Si Young Song, Miseon Kang

**Affiliations:** Yonsei University, Seoul, Republic of Korea; Yonsei University, Seoul, Republic of Korea; Kyung Hee University, Seoul, Republic of Korea; Yonsei University, Seoul, Republic of Korea

## Abstract

This study aimed to identify subtypes of Social Network Service (SNS) use that affect self-esteem by applying a person-centered approach. The study also aimed to compare the SNS use subtypes and their associations with self-esteem between young and middle-aged adults in Korea. The sample included 2,572 young adults (18-44 years old) and 1,790 middle-aged adults (45-64 years old) drawn from the Korea Media Panel, a nationally representative dataset. Self-esteem was measured by the sum of ten questions from Rosenberg’s Self-Esteem Scale. Four indicators of SNS use (e.g., “How often do you check other people’s posts (feed)?”) were used to perform a 3-step Latent Profile Analysis, which accounts for classification errors. The results of the Latent Profile Analysis showed 5 and 4 subtypes of SNS use for young and middle-aged adults, respectively, depending on the frequency of active and passive usage. Overlapping subtypes include ‘passive-low’, ‘active-low’, ‘moderate’, and ‘high-active’ users. Young adults had one distinct subtype with a very high frequency of passive usage (checking others’ posts and liking them) but a very low frequency of active usage (sharing content with others). The results also revealed that those with more frequent use in both active and passive use showed the lowest self-esteem in both young and middle-aged groups. This study supports previous findings suggesting that frequent SNS use has negative impacts on self-esteem, even for middle-aged adults. Based on these results, digital literacy programs for middle-aged adults should cover healthy ways to use SNS.

